# Threat of shock promotes passive avoidance, but not active avoidance

**DOI:** 10.1111/ejn.15184

**Published:** 2021-03-24

**Authors:** Aida Helana Binti Affandi, Alexandra C. Pike, Oliver Joe Robinson

**Affiliations:** ^1^ Anxiety Lab, Neuroscience and Mental Health Group Institute of Cognitive Neuroscience University College London London UK

## Abstract

Anxiety and stress are adaptive responses to threat that promote harm avoidance. In particular, prior work has shown that anxiety induced in humans using threat of unpredictable shock promotes behavioral inhibition in the face of harm. This is consistent with the idea that anxiety promotes *passive avoidance*—that is, withholding approach actions that could lead to harm. However, harm can also be avoided through *active avoidance*, where a (withdrawal) action is taken to avoid harm. Here, we provide the first direct within‐study comparison of the effects of threat of shock on *active* and *passive* avoidance. We operationalize passive avoidance as withholding a button press response in the face of negative outcomes, and active avoidance as lifting/releasing a button press in the face of negative outcomes. We explore the impact of threat of unpredictable shock on the learning of these behavioral responses (alongside matched responses to rewards) within a single cognitive task. We predicted that threat of shock would promote both active and passive avoidance, and that this would be driven by increased reliance on Pavlovian bias, as parameterized within reinforcement‐learning models. Consistent with our predictions, we provide evidence that threat of shock promotes passive avoidance as conceptualized by our task. However, inconsistent with predictions, we found no evidence that threat of shock promoted active avoidance, nor evidence of elevated Pavlovian bias in any condition. One hypothetical framework with which to understand these findings is that anxiety promotes passive over active harm avoidance strategies in order to conserve energy while avoiding harm.

## Graphical abstract

In this study, we provide the first direct within‐study comparison of the effects of threat of shock on *active* and *passive* avoidance. We operationalize passive avoidance as withholding a button press response in the face of negative outcomes, and active avoidance as lifting/releasing a button press in the face of negative outcomes. Participants were *less* accurate when *pressing* to avoid losing under threat of shock (than when they were required to press to win). By contrast, there was no valence effect on lift trials under threat of shock. We interpret this as threat of shock promoting passive avoidance (i.e., no go to avoid losing), but having no effect on active avoidance (i.e., lift to avoid losing).

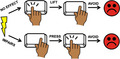

## 1 INTRODUCTION

The psychological and physiological responses to uncertain, unclear, and potentially imagined stressors or threats—defined here as ‘anxiety’—are key to the dynamics of mood and anxiety disorders (Adamec et al., 1998; Blanchard & Blanchard, 2008). One core anxiety response is avoidance (Meacham & Bergstrom, 2016), which may be defined as the adaptive, harm‐reducing, act of creating physical or psychological distance from a threat. Many people, for instance, avoid situations which have a high potential for social embarrassment, such as sitting in the front row at a live comedy event. However, excessive avoidance might result in a socially anxious individual becoming housebound in an attempt to avoid all potential social embarrassment.

Avoidance can, moreover, be divided into at least two basic tendencies (Davidson, 1998; Miller, 1944): *active* or *passive* (Carver, 2006; Schlund et al., 2010). Active avoidance is when someone ‘does something’ to avoid a particular outcome (e.g., walking down a different aisle in a supermarket to avoid having a social encounter) whereas passive avoidance is when someone ‘does *not* do something’ to avoid the outcome (e.g., staying at home instead of going to the supermarket). In other words, *passive avoidance* can be defined as *inhibiting approach* while *active avoidance* can be defined as *promoting withdrawal*.

Stress/anxiety induced by threat of shock (Mkrtchian et al., 2017; Mkrtchian et al., 2017; Robinson et al., 2012, 2013) has been shown to promote behavioral inhibition and passive avoidance (Mkrtchian, Aylward, et al., 2017; Robinson et al., 2013; Torrisi et al., 2018) (Note that the threat of shock is variously referred to as inducing ‘anxiety’ or ‘stress’ depending on the context, but is perhaps best referred to simply by description (response to unpredictable electrical shocks) ‐ to avoid confusion caused by field‐specific definitions of ‘anxiety’ or ‘stress’). In particular, Mkrtchian, Aylward, et al., (2017) measured avoidance using a Go/No‐Go task, in which participants responded by either pressing (‘go’/approach) or not pressing (‘no‐go’/do not approach) a button, under threat or safe conditions. Overall, participants showed what is referred to as ‘Pavlovian bias’: ‘go’ (approach) was preferred in the context of winning while ‘no‐go’ was preferred when avoiding loss. Specifically, participants were less accurate when asked to ‘go’ to avoid harm versus ‘no‐go’ to avoid harm, which was interpreted as a reliance on passive avoidance. Moreover, this bias (as parameterized by a reinforcement learning model) was exacerbated by threat in participants with mood and anxiety disorders. Therefore, this study (Mkrtchian, Aylward, et al., 2017) indicated that threat of shock affected avoidance behavior in anxious individuals by amplifying a prepotent Pavlovian bias to avoid threats. In a slightly different task designed to measure the same avoidance behavior but using response time distributions as the dependent variable, threat of shock was also shown to promote inhibition of approach responses in the face of harm (Mkrtchian, Roiser, et al., 2017). Taken together, both studies suggested that anxiety promotes avoidance, but the design of both tasks was restricted to inhibiting approach (i.e., ‘press’) responses and, as such, can be thought of as primarily demonstrating that threat of shock can promote *passive avoidance*.

To explore *active* avoidance, Gorka et al., (2016) used a slightly different design to explore the association of withdrawal responses with (predictable) shocks. In this study, participants were required to hold down a keyboard button and lift their finger (i.e., actively withdraw) whenever they detected an avoidance cue. They found that threat of shock promoted finger lifting, or, in other words, *active* avoidance. However, the design of this task was such that shocks could be avoided through accurate task performance, so increased withdrawal was actually incentivized by the task design. In the Mkrtchian et al. work on passive withdrawal, the shocks were *not* contingent on task performance, so behavior was not confounded by attempts to reduce shock‐related threat. The impact of anxiety/stress induced by performance‐unrelated threat of unpredictable shock on active avoidance—as conceptualized by finger lifting—has not, to our knowledge, been explicitly tested.

The purpose of the present study was therefore to directly compare the impact of threat of shock on passive and active avoidance, in the same paradigm. We operationalized passive avoidance as withholding a button press in the face of negative outcomes, and active avoidance as releasing/lifting a button press in the face of negative outcomes. Specifically, we used the same task and anxiety induction as Mkrtchian, Roiser, et al., (2017), but expanded the press/no‐press approach (i.e., passive avoidance) condition to also encompass the lift/no‐lift withdrawal (i.e., active avoidance) condition from Gorka et al., (2016). Studying both types of avoidance (active and passive) within the same paradigm will allow us to *directly* compare participants’ accuracy and response times when the required response is an active withdrawal (releasing a key) compared to a passive withdrawal (refraining from pressing any key). This will enable us both to test whether threat of shock affects both of these responses, and to see whether these responses are affected in comparable or quantitatively different ways.

We predicted that threat of shock would promote both passive and active avoidance. Specifically, we predicted that threat of unpredictable shock would make it easier for individuals to both withhold a button press (passive avoidance) and lift to release a button press (active avoidance) to avoid punishment (Gorka et al., 2016; Mkrtchian, Aylward, et al., 2017; Mkrtchian, Roiser, et al., 2017). We predicted, moreover, that this would be driven by elevated reliance on participants’ Pavlovian bias, as parameterized by previously used reinforcement learning models (Mkrtchian, Aylward, et al., 2017), for both task (press/lift) conditions.

## 2 METHODS

All scripts and data for this experiment can be found online at https://osf.io/wc3mu/.

### 2.1 Participants

A number of 59 healthy participants were recruited from the UCL Psychology Subject Pool SONA system database. The number of subjects was determined by an a priori power analysis in G*Power (Faul et al., 2007). Based on prior work (Mkrtchian, Roiser, et al., 2017) we performed a power analysis based on a Cohen's d_z_ effect size of 0.4 for the simplest possible comparison across within‐subject conditions. A matched paired *t* test requires *N* = 52 participants at the 0.05 alpha level with 80% power (*N* = 59 assuming ~ 10% drop‐off or missing data). Participants were only eligible if they reported no psychiatric, neurological, cardiac, or endocrine problems and were not taking any psychotropic medication (as per an email screen). Participants were reimbursed £7.50 for taking part in this study (which was not contingent on task performance), which lasted ~ 1 hr. Their written informed consent was obtained prior to their participation. This study obtained ethical approval from the UCL Research Ethics Committee (Project ID Number : 6198/001/2019).

### 2.2 Experimental design

The experiment consisted of three parts: (1) the State‐Trait Anxiety Inventory (STAI; Spielberger et al., 1983) questionnaire, then the Approach‐Withdrawal task which consisted of two parts, (2) the Practice Task, and (3) the Main Task.

### 2.3 Approach‐withdrawal task

The approach‐withdrawal task is based on a combination of the active avoidance of signal threat (AAST) task from (Gorka et al., 2016) and the Go/No‐Go task from (Mkrtchian, Aylward, et al., 2017). It was programmed using MATLAB (2017a, The MathWorks, Natick, MA) and presented using the Cogent 2000 Toolbox (v1.32, www.vislab.ucl.ac.uk).

#### 2.3.1 Practice task

The purpose of the practice task presented before the main task was to allow the participants to learn how to perform eight actions: (1) Press to Win, (2) Don't Press to Win, (3) Lift to Win, (4) Don't Lift to Win, (5) Press to Avoid Losing, (6) Don't Press to Avoid Losing, (7) Lift to Avoid Losing, and (8) Don't Lift to Avoid Losing. The practice consisted of 24 trials, with each action repeated three times (order randomized). During the practice task, explicit instructions were given, which distinguished it from the main task (see Table 1).

**TABLE 1:** List of the eight actions with the corresponding instruction given to the participant during training

Action	Instructions
Press To Win	“Press the spacebar as soon as the START cue appears”
Don't Press To Win	“Do NOT press any key at any cue”
Lift to Win	“Press and hold the spacebar at the GET READY cue and lift the spacebar as soon as the START cue appears”
Do Not Lift to Win	“Press and hold the spacebar at the GET READY cue and do NOT lift the space bar at the START cue.”
Press to Avoid Losing	“Press the spacebar as soon as the START cue appears”
Do Not Press to Avoid Losing	“Do NOT press any key at any cue”
Lift to Avoid Losing	“Press and hold the spacebar at the GET READY cue and lift the spacebar as soon as the START cue appears”
Do Not Lift to Avoid Losing	“Press and hold the spacebar at the GET READY cue and do NOT lift the spacebar at the START cue.”

**FIGURE 1:**
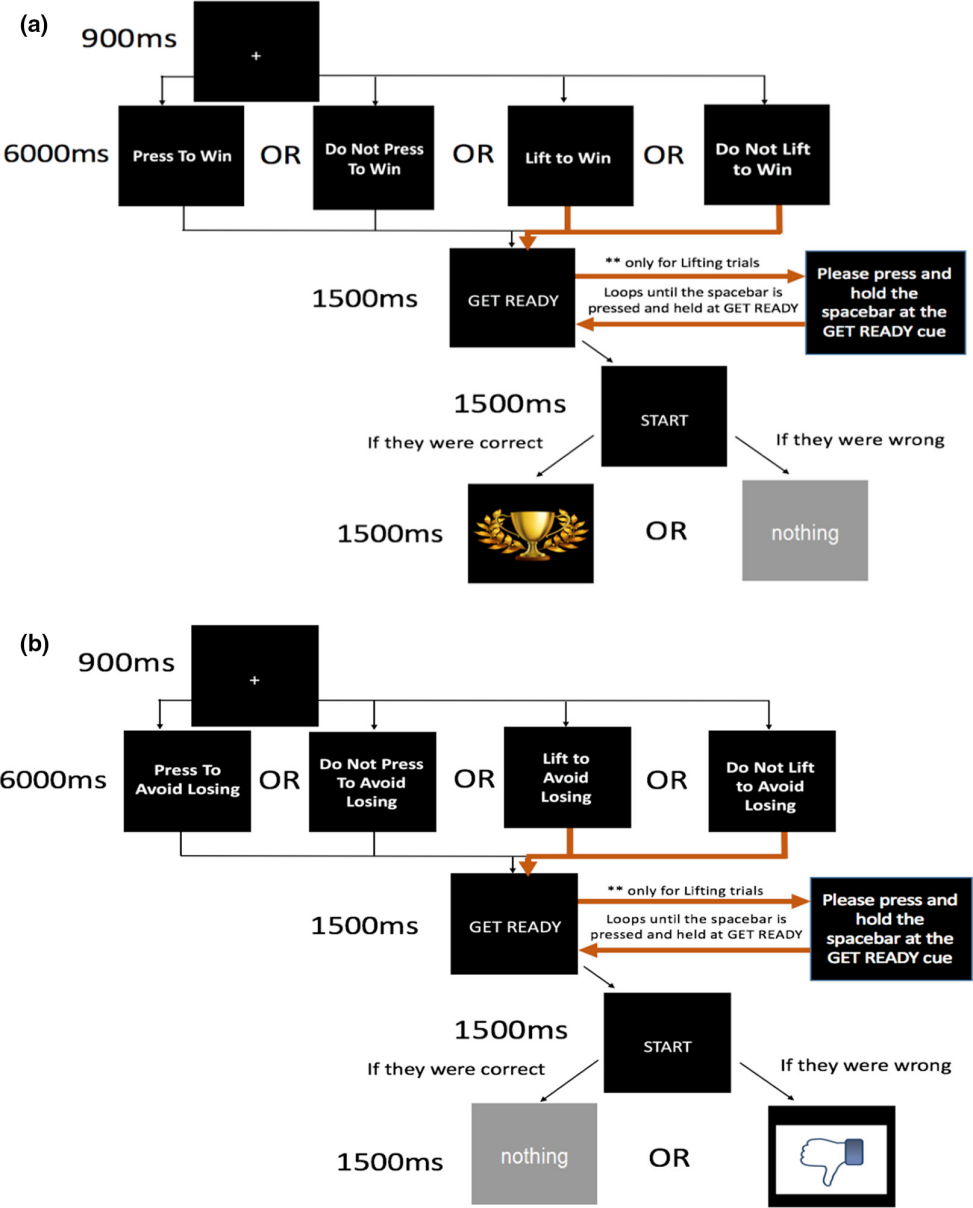
Diagram of practice task for the ‘win’(A) and ‘avoid losing’(B) trials. Each trial began with a 900 ms display of the fixation cross followed by a 6000 ms display of the action names coupled with the instructions (not shown here). Subsequently, the ‘Get Ready’ cue was displayed. For ‘press’ trials, the task moved on to ‘Start’ right afterwards, for the participants to act on the instructions. For the ‘lift’ trials, ‘Start’ was only shown if the participant pressed and held the spacebar down at the ‘Get Ready’ cue. Otherwise, the participant was continuously prompted to do so in a loop. For the ‘win’ trials, participants were shown a trophy if they were correct and ‘nothing’ if wrong. Conversely, for the ‘avoid losing’ trials, participants were shown ‘nothing’ if they were correct and the ‘thumbs‐down’ picture if they were wrong

These instructions were read verbally to each participant to aid understanding. The participant was instructed to press the spacebar once they were ready to start. A visualization of the practice task is shown in Figure 1. Specifically, a fixation cross was displayed for 900 ms followed by a 6000 ms presentation of the name of the action they had to perform. Following this, “Get Ready” followed by “Start” was each displayed for 1500 ms.

For the “Press to Win/Avoid Losing” trials, participants were required to press the space bar as soon as “Start” was presented in order for their response to be classed as ‘correct’. For the “Do Not Press To Win/Avoid Losing” trials, any press of the spacebar during either cue would result in the response being classed as ‘incorrect’. During the lift trials, “Lift to Win/Avoid Losing” and “Do Not Lift to Win/Avoid Losing”, participants were required to press and hold the spacebar at “Get Ready” and subsequently lift or (not) at “Start” depending on the instructions. If they did not press and hold the spacebar down at “Get Ready” for a lift trial they would be prompted to do so in a loop, such that the trial would not progress until they pressed and held the spacebar. Once the “Start” cue was displayed, participants had up to 1500 ms to make their response.

After the action, for the outcomes in the “win” trials, the participants would see a picture of a trophy if they performed the right action, and they would see the word “nothing” otherwise. For the “avoid losing” trials, the participants would see “nothing” if they performed the right action (meaning they had avoided a ‘loss’ outcome) or they would see a ‘thumbs‐down’ picture, signifying loss. The outcomes were displayed for 1500 ms. This was repeated for 24 trials. To ensure that participants were certain of the correct response, the outcomes were not probabilistic. Carrying out the practice task took approximately 5 min for all participants.

#### 2.3.2 Main task

After the participants completed the practice task and were comfortable with performing the eight actions, they moved onto the main task. Participants completed 30 of each of the eight trial types described in Table 1, under alternating conditions of safe and threat (see below ‐ order counterbalanced across participants), making up to a total of 240 trials for each participant. The 240 trials were divided into six blocks of 40 trials. The main task took approximately 30 min to perform.

**FIGURE 2:**
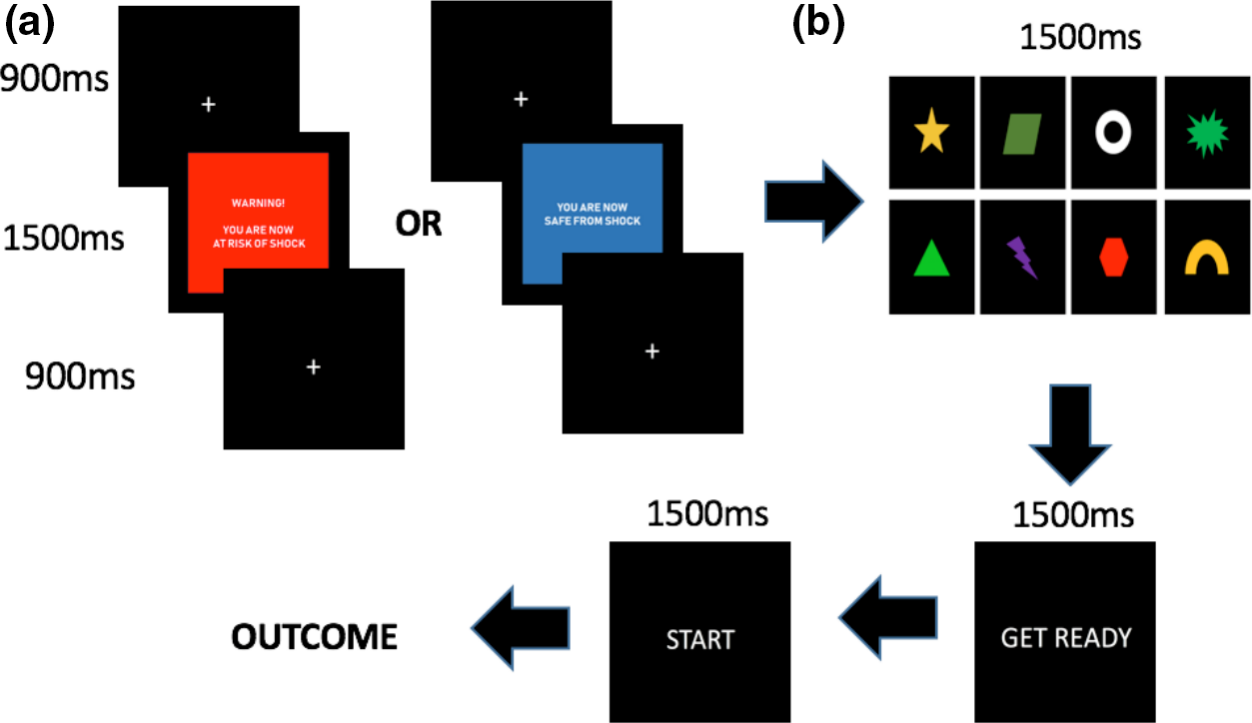
Main Task Paradigm. (a) Before the start of each block, participants were informed of the nature of each block, whether shock (left) or safe (right). (b) The eight shapes, each of which had a different associated action, randomized for each participant. One shape was shown for each trial, which progressed on to the “Get Ready” and “Start” cues (in that order). Outcomes were the same as for the practice task, except that they had a probabilistic nature

The eight trial types were predicted by eight shapes: star, ring, triangle, lightning bolt, explosion, parallelogram, arch, and hexagon (counterbalanced across participants; see Figure 2) and participants were instructed to learn which shape required which action, via trial‐and‐error. Each shape was presented for 1500 ms followed by the cues “Get Ready”, “Start” and the outcome with the same timings as the practice task. Participants were told that they began each block with 40 points. The trophy outcome meant that they earned 1 point, and the thumbs down outcome resulted in a loss of 1 point. At the end of each block, they were shown their total points for that block. Before the start of each block, participants were given the option to take a self‐paced break, after which they could continue the task by pressing the spacebar.

#### 2.3.3 Threat of shock (i.e., anxiety/stress) manipulation

Unpredictable shocks, not dependent on performance, were delivered using a Digitimer DS5 Constant Current Stimulator (Digitimer Ltd, Welwyn Garden City, U.K.). This device delivered highly unpleasant but non‐painful shocks (Schmitz & Grillon, 2012) via two electrodes attached on the ventral side of the participants’ nondominant wrists. Shock level was determined via the same shock work‐up procedure as Mkrtchian, Roiser, et al., (2017) (prior to carrying out the Practice Task). To avoid habituation, participants were given no more than five shocks and rated each of them on a scale of 1 (*barely felt it*) to 5 (*unbearable*). The shock level chosen for the main paradigm was level at which the participants rated the shock as 4 out of 5 in unpleasantness.

The six blocks were divided into two types, safe and shock, the order of which was counterbalanced across participants. Before the start of each block, participants were informed of the type of block they were in on screen (Figure 2). Trials in the safe block had a blue background, and no shocks were delivered during this block. Trials in the shock block had a red background. One shock was given for each shock block in a probabilistic manner (three shocks in total for the whole task): 50% of the time, shocks were given in the 12th trial of the block and for the other half of the time, shocks were given in the 28th trial of the block. Delivery of a shock was during a fixation cross buffer accompanied with a black background for 1500 ms.

Following the main task, participants were asked to rate how anxious they had felt in the safe condition and the shock condition, and then how stressed they had felt in the safe condition and the shock condition on a 5 point scale (*1 – not at all, 2‐slightly anxious/stressed, 3‐moderately anxious/stressed, 4‐very anxious/stressed, 5‐extremely anxious/stressed*). This was to confirm that this manipulation had the intended effect of increasing stress/anxiety levels.

Unlike the practice task, where the outcomes are direct consequences of the actions, the main task had probabilistic outcomes, as in Mkrtchian, Aylward, et al., (2017). In the main task, 80% of the time the participants would receive the expected outcome, while 20% of the time participants would receive the opposite outcome. This was explicitly explained to the participants, who were instructed to take actions based on the most likely outcome.

### 2.4 Statistical analysis

Accuracy and reaction times (RT) were analyzed using repeated‐measures ANOVAs run in JASP (version 0.9.2) and the afex package in R. The factors included in a full factorial repeated measures ANOVA were: Threat of Shock (TOS; Safe vs. Shock), Action (Press versus. Lift), Instruction (Do versus. Don't), and Valence (Win vs. Avoid Losing). Block is an operationalization of time, and was included in a separate full factorial ANOVA (factors Block (1:6), Action (Press vs. Lift), Instruction (Do vs. Don't), Valence (Win vs. Avoid Losing)) to determine if there were any learning effects. Where the assumption of sphericity was violated, a Greenhouse‐Geisser correction was applied. Paired sample *t* tests were carried out to dissociate the simple effects driving significant interactions in the ANOVA (corrected for multiple comparisons using False Discovery Rate (FDR) correction).

### 2.5 Computational modeling

We fit four Reinforcement Learning models using Bayesian hierarchical Markov chain Monte Carlo parameter fitting implemented by the hBayesDM toolbox (Ahn et al., 2017). These models, described comprehensively elsewhere (Guitart‐Masip et al., 2012), comprise nested Rescorla Wagner models (Rescorla & Wagner, 1972) with a learning rate (*ep*; m1/m2/m3/m4) and either one sensitivity (*rho*; m1/m2/m3) or separate sensitivity parameters for rewards and punishments (*rhoRew/rhoPun*; m4), plus additional parameters including noise (*xi*; m1/m2/m3/m4), a bias toward making actions (vs. doing nothing *b*; m2/m3/m4), and a Pavlovian bias to approach reward (*pi*; m3/m4).

We fit all four models to participants’ choice behavior (go or no‐go) for each of the four task conditions separately: Threat Press; Threat Lift; Safe Press; Safe Lift. We determined the overall winning model by summing fit indices (Leave One Out Information Criterion; LOOIC) across all four conditions. We next tested the fit for this winning model in each condition by simulating task performance for each individual and comparing it to their actual task performance. Having established posterior predictive fit to the data, we finally ran inference on parameters across task conditions. Specifically, we compared the parameters from the winning model across task conditions using the 95% highest density intervals (HDI) of the difference in hyperparameters. Where this difference did not overlap zero, we considered there to be a credible difference on parameter values between the task conditions (Ahn et al., 2017; Kruschke, 2014). We calculated the overall condition by action interaction by calculating a ((threat press – safe press) – (threat lift – safe lift)) difference score, as well as simple effects by computing all four pairwise comparisons.

## 3 RESULTS

### 3.1 Participants

The final sample consisted of 59 participants (29 female; age range = 19 to 61; mean = 30±11). Participants had a mean STAI state anxiety score of 33 ± 10 and a mean STAI trait anxiety score of 37 ± 10.

### 3.2 Manipulation check

There was a significant increase in the anxiety ratings from the safe (mean (M)=1.5, standard deviation (*SD*)=0.8)) to threat (*M* = 3.0, *SD* = 1.1) conditions; (*t*_58_ = 10.8, *p* <0.001, 95% CI = 1.2:1.8) and a significant increase in the stress ratings from the safe (*M* = 1.7, *SD* = 0.76) to threat (*M* = 3.0, *SD* = 0.94) conditions (*t*_58_ = 9.54, *p* <0.001, 95% CI = 1.0:1.5). None of these post task ratings correlated with STAI state anxiety (all *r* < 0.16, *p* >0.24), indicating that the task‐induced anxiety was independent from dispositional anxiety (n.b. STAI trait and state scales correlate *r* = 0.8).

### 3.3 Accuracy

All significant main effects and interactions from two full factorial repeated measures ANOVAs are detailed below. The main ANOVA had four factors: TOS (Safe vs. Shock), Action (Press vs. Lift), Instruction (Do vs. Don't), and Valence (Win vs. Avoid Losing). The second ANOVA replaced the TOS factor with block as a factor (1:6). Non‐significant interactions (*p* >0.08) can be found in the open materials. Note that collapsed performance is substantially greater than chance level of 0.25 (for four actions: do press, don't press, do lift and don't lift) within each condition indicating that participants were able to (at least partially) learn the stimulus‐outcome contingencies.

### 3.4 Main effect of valence

**FIGURE 3:**
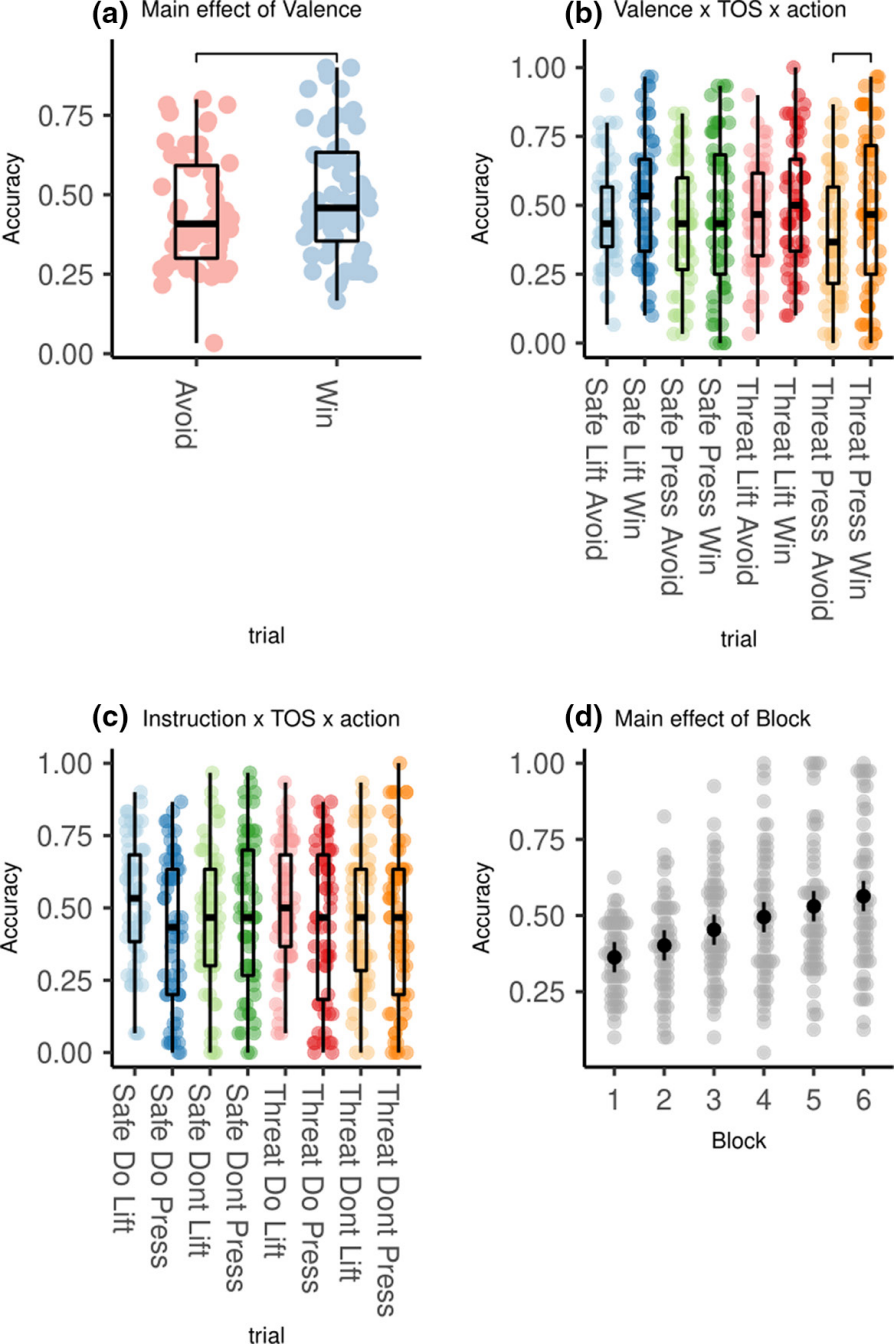
Graphs summarizing results of repeated measures ANOVA looking at effect of Valence (Win vs. Avoid), TOS (TOS vs. Safe), Action (Press vs. Lift), Instruction (Do vs. Don't) on accuracy). (a) The main effect of valence is demonstrated, where accuracy is significantly higher when winning compared to when avoiding loss. (b) Valence × TOS × Action interactions where TOS Press Win has a significantly higher accuracy then TOS Press Avoid. (c) Instruction × TOS × Action interaction, where there was a significant crossover interaction between TOS and instruction in the Press (*F*_1,109.17_ = 11.5, *p* <0.001), but not Lift (*F*_1,109.17_ = 05, *p* =0.5) conditions. (d) A main effect of Block is also seen on a second ANOVA replacing TOS with Block (1:6), where accuracy increases significantly between Blocks 1 to 6, signifying that the task is learnt appropriately with time. Brackets indicate significant FDR‐corrected paired sample *t* tests for A:C – significant changes for every contrast apart from block 1 vs. 2 are omitted from D

There was a main effect of valence (*F*_1,58_ = 0.73, *p* =0.009, $\eta_{p}^{2}$ **= **0.11), driven by increased accuracy when responding to win versus avoiding loss (Figure 3a).

### 3.5 Valence × TOS × action interaction

There was a significant interaction between valence, TOS and action (*F*_1,58_ = 5.13, *p* = 0.027, $\eta_{p}^{2}$ = 0.081; Figure 3b). Participants were *less* accurate (*t*_117_ (*df* = 117) = 3.0, *p_(FDR)_* = 0.019, dz = 0.32) when *pressing* to avoid losing under threat (*M* = 0.4, *SD* = 0.2) than when they were required to press to win (*M* = 0.49, *SD* = 0.027)—that is, an effect of valence on the press trials under threat. By contrast, there was no valence effect on lift trials under threat of shock (*t*_117_ *= *1.4, *p_(FDR)_* = 0.51, dz = 0.36). Moreover, there was no significant effect of valence in either the press (*t*_117_ = 1.2, *p_(FDR)_* = 0.64, dz = 0.3) or lift (*t*_117_ = 1.81, *p_(FDR)_* = 0.27, dz = 0.47) trials under safe conditions (Figure 3b). In other words, threat attenuated the ability to press to avoid, perhaps due to an increased reliance on *passive avoidance* (but not active avoidance, as evidenced by a lack of effect of valence on the lift conditions).

### 3.6 Instruction × TOS × Action interaction

There was also a significant interaction between instruction, TOS, and action (*F_1,58_* = 11, *p* =0.001, $\eta_{p}^{2}$ = 0.162; Figure 3c). Notably, none of the corrected post hoc *t* tests were significant, but there was a significant crossover interaction between TOS and instruction in the Press (*F*_1,109.17_ = 11.5, *p* <0.001), but not Lift (*F*_1,109.17_ = 05, *p* =0.5) conditions.

### 3.7 Main effect of block

A main effect of block was also observed in the second ANOVA, which replaced TOS with block as a factor (*F*_2.96, 171.69_ = 32, *p* <0.001, $\eta_{p}^{2}$ **= **0.36). Paired *t* tests carried out post‐hoc showed that this main effect was driven by a significant increase in accuracy as blocks progressed for all transitions (all p_(FDR)_ < 0.001, *d* > 1.2) after the transition from block 1–2 (Figure 3d). This demonstrates that participants were able to learn the stimulus action associations, and justifies the reinforcement learning analysis (see computational modeling section in the supplementary materials). There was also an interaction between block and valence (*F*_4.03, 233.82_ = 3.7, *p* =0.006, $\eta_{p}^{2}$ = 0.06), as individuals initially had a higher accuracy for win trials, but by the final block accuracy was equal for win and avoid trials (Figure S1). There were no other interactions between block and effects of interest (all *p* >0.08).

### 3.8 RT

There was a significant main effect of block driven by increasing speed of response over blocks (*F*_1.858,52.013_ = 0.263, *p* <0.001, $\eta_{p}^{2}$ = 0.263) but no other interaction or main effects (all *p* >0.21).

### 3.9 Computational modeling

All modeling data and results are available as a Markdown document (https://osf.io/wc3mu/) and in the supplementary materials. Briefly, the winning model was model 4, which simulations demonstrated was also a good fit to the data (Figure S2). The only parameter which showed a credible difference across conditions was the noise parameter (Table S1), for which simple effects demonstrated increased behavioral noise in the lift (relative to press) action under the safe condition only (Figure S3).

## 4 DISCUSSION

In this study, we added separate approach (press) and withdrawal (lift) actions to a previously used Go/No‐Go task (Mkrtchian, Aylward, et al., 2017) and explored the interaction between these actions and threat of shock (TOS). Consistent with our predictions, we showed that TOS promoted passive avoidance. Specifically, we showed that threat of shock modulated the performance of actions (Valence × TOS × Action) such that individuals were selectively impaired at approaching (press) to avoid losses (relative to pressing to gain wins) under threat (but not safe) conditions, perhaps due to increased passive avoidance under threat. By contrast, we did not find evidence to support our prediction of increased active avoidance under threat.

Consistent with our first hypothesis, we found some evidence of increased reliance on passive avoidance processes under threat of shock. Specifically, we observed a Valence × TOS × Action interaction, which was driven by the *attenuation by threat* of participants’ accuracy in pressing to avoid losses (relative to pressing to gain a win; see Figure 5b). We would argue that this is likely due to participants’ reduced ability to press in the face of punishments, rather than an improved ability to press in the face of rewards. In other words, we argue that participants found it harder to approach in the face of punishments when anxious. One explanation for this is that threat of shock increases reliance on passive avoidance in the face of punishment. This is consistent with anxiety promoting harm avoidance through passive avoidance (Mkrtchian, Aylward, et al., 2017; Mkrtchian, Roiser, et al., 2017).

Inconsistent with our second prediction, however, a similar effect was not seen for the active withdrawal (i.e., lift) condition of this task. Taken together with the effect on the press condition, this suggests that anxiety selectively reduces an individual's tendency to approach losses (which is adaptive) but does not also promote their tendency to withdraw from losses (which would also be adaptive), at least as framed in the current task. In other words, threat of shock promotes passive avoidance in the face of punishments, but has no specific effect on active avoidance. If this is the case, perhaps it is because anxiety favors harm avoidance mechanisms that conserve (i.e., passive), rather than expend (i.e., active), energy, as has been suggested previously (Bach, 2015; Patzelt et al., 2019; Roskes et al., 2013). Interestingly, this finding is inconsistent with previous work on active avoidance, which demonstrated increased lift responses in the face of threats (Gorka et al., 2016). However, this might be explained by the fact that the threat in this previous paradigm was performance‐dependent, such that improved performance led to fewer shocks. In the current task, performance was independent from shocks, which allowed us to explore the impact of anxiety *in general* on performance, rather than how it may incentivize performance. Perhaps where performance will reduce threat it is adaptive to expend energy, but where performance has no impact on threat then it is adaptive to conserve energy.

Finally, inconsistent with our third hypothesis, this study did not detect an effect of task condition or threat on the Pavlovian bias parameter in the reinforcement learning modeling. Note that the winning model *did* contain the Pavlovian bias parameter. So, consistent with multiple prior studies (Albrecht et al., 2016; Millner et al., 2018; Moutoussis et al., 2018; Ousdal et al., 2018; Raab & Hartley, 2020), Pavlovian bias does likely play a key role in the interaction between action and valence on task performance. It is simply that the influence of this parameter did not change as a function of pressing/lifting or threat/safe condition. This calls into question whether any *anxiety‐induced* avoidance bias is driven by reliance on prepotent Pavlovian mechanisms (at least as parameterized in our reinforcement learning model). However, in Mkrtchian, Aylward, et al., (2017) this bias was only revealed in patients with anxiety disorders undergoing threat of shock (and was not seen under threat of shock in the controls). As such it may be that TOS only elevates reliance on Pavlovian biases in clinically anxious individuals. The present finding is actually closer to that observed in Mkrtchian, Roiser, et al., (2017), in which threat of shock leads to selective speeding on ‘press to avoid’ trials in a healthy control sample. In this paper, this bias was interpreted as increased *inhibition* under threat press trials, which would manifest as increased errors on the present task. Perhaps, therefore, threat of shock promotes inhibitory processes which are independent from Pavlovian biases. Alternatively, Pavlovian biases may not be fully captured by the parameters in this model: our data are available online, so that different models can be tested in the future.

Despite no evidence for elevated active avoidance, we did observe some evidence that generic active withdrawal behavior was influenced by threat. Specifically, we saw an Instruction x TOS × Action interaction driven by a TOS × Instruction interaction in the Press, but not Lift condition. None of the individual pairwise comparisons were significant, which was unexpected, since it was thought that a specific tendency to withdraw (i.e., Lift) in the face of punishments would arise under the TOS condition, as seen in Gorka et al., (2016), where withdrawal was significantly increased when the participants anticipated receiving the shocks. Although, as previously mentioned, in the Gorka task shocks were contingent on performance, which was not the case in this task. In this context, it is important to highlight a key task‐design limitation, which is that at the start of lift trials, participants were prompted to depress the spacebar first, and the trial would not progress without their doing so. This means participants could learn that prompted trials were lift trials. This may have made the condition easier, and hence less subject to gradual reinforcement learning. Alternatively, this trial type may have actually been *harder* because it entailed making two actions (press and then lift) rather than just one. Task difficulty differences associated with the lift trials do not explain why lift effects were specific to the safe condition, but it is possible that these differences affected our ability to detect withdrawal effects, and should be addressed in future task designs by, for example, using a different method of measuring approach/withdrawal, such as a joystick or mouse movements. Overall however, we found no clear evidence that active avoidance was, as predicted, promoted by induced anxiety in this task design. Another possibility is that the presence of rewards in this task changes the context (as well as increasing the number of trial types to learn)—future work might replicate this design but simplify it into a punishment only version.

In sum, one way to consider these effects is that anxiety switches the system into a passive avoidance mode; perhaps as a means of minimizing harm while maximizing energy conservation. This is consistent with the clinical picture, whereby some anxious individuals end up housebound to (passively) avoid potential stressors (e.g., social situations). Future work should consider whether performance on this abstract task is correlated with these real‐life passive avoidance symptoms in clinically relevant anxiety. In particular, determining whether increased passive, but *not* active, avoidance is key to anxious symptoms in some patients may help refine our treatment strategies for these debilitating disorders.

## 5 CONFLICT OF INTEREST STATEMENT

The funder had no role in the study design, collection, analysis, or interpretation of data. OJR's MRC senior fellowship is partially in collaboration with Cambridge Cognition (who plan to provide in‐kind contribution) and he is running an investigator‐initiated trial with medication donated by Lundbeck (escitalopram and placebo, no financial contribution). He also holds an MRC‐Proximity to discovery award with Roche (who provide in‐kind contributions and have sponsored travel for ACP) regarding work on heart‐rate variability and anxiety. He has also completed consultancy work on affective bias modification for Peak and online CBT for IESO digital health. OJR sits on the committee of the British Association of Psychopharmacology. ACP and AHBA declare no other conflicts of interest.

## AUTHOR CONTRIBUTIONS

OJR conceived the project. AHBA collected and analyzed the data, with supervision by OJR and ACP. All authors drafted the manuscript and revised it. All authors have approved the final article.

### PEER REVIEW

The peer review history for this article is available at https://publons.com/publon/10.1111/ejn.15184.

## Supporting information

Supplementary Material

## Data Availability

All scripts and data for this experiment can be found online at https://osf.io/wc3mu/.
