# Supplementary material for: Threat of shock promotes passive avoidance, but not active avoidance
Source: Eur J Neurosci. 2021 Mar 24;55(9-10):2571–80. doi: 10.1111/ejn.15184 (PMC12086743; doi:10.1111/ejn.15184)
Supplement: Supplementary file 1 — Supplementary Material [file EJN-55-2571-s001.pdf]

Supplement

Post hoc Trait anxiety effects

There was no main effect of Trait anxiety on performance accuracy ($p=0.46$) and it did not interact with any of the reported interactions (all $p>0.09$) indicating that the effects were driven by the anxiety induction rather than dispositional anxiety.

Post hoc Order effects

There was no main effect of the order of safe/threat blocks on performance accuracy ($p=0.76$) and this did not interact with any of the reported interactions (all $p>0.064$). There was an order by condition effect ($F(1,57)=21$, $p<0.001$ $\eta_p^2=0.27$) driven by increased accuracy overall in the safe conditions relative to threat conditions when the first block was threat ($t_{57}=3.9$, $p_{(FDR)}=0.001$, dz=1), but not when it was safe ($t_{57}=0.5$, $p_{(FDR)}=0.96$, dz=0.12).

Post hoc Correlation between behavior and manipulation check

There was no significant correlation between the observed behavioural effect (Threat Press Win – Threat Press Avoid) and the change in either anxiety ($r_{(57)}=-0.1$, $p=0.5$)) or stress ($r_{(57)}=-0.02$, $p=0.9$) manipulation check scores.

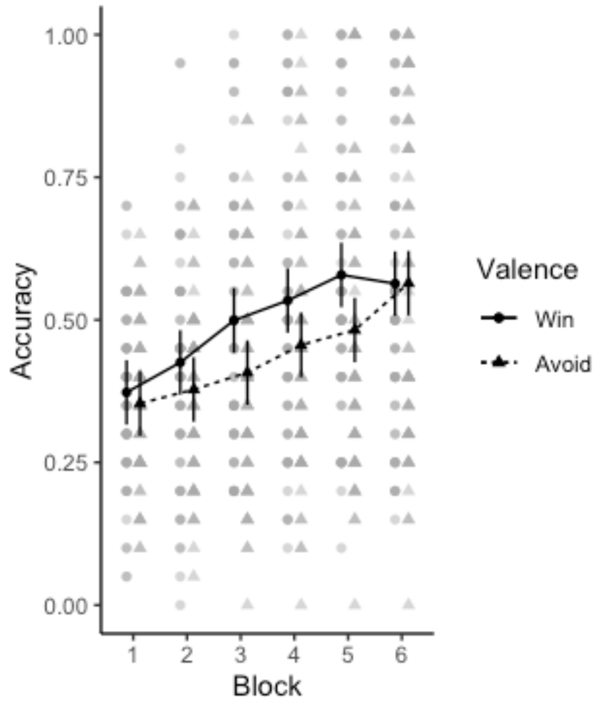

Figure S1 The interaction between block and valence demonstrating initial faster learning for Win relative to Avoid trials.

Computational modelling

Model fitting (Figure 4A) identified m4 (i.e. Rescorla Wagner learning, with the following parameters: learning rate (*ep*) + separate reward (rhoRew) and punishment (rhoPun) sensitivities + noise (*xi*) + action bias (*b*) + pavlovian bias (*pi*)) as the winning model (LOOIC=10231), which was substantially better ($logBF_{10}$=27) than the next best model (m3: the same as m4, but with a single sensitivity parameter rather than separate sensitivity parameters for reward and loss; LOOIC=10355). Simulations (Figure S1B:F) demonstrated excellent posterior predictive fits for this model.

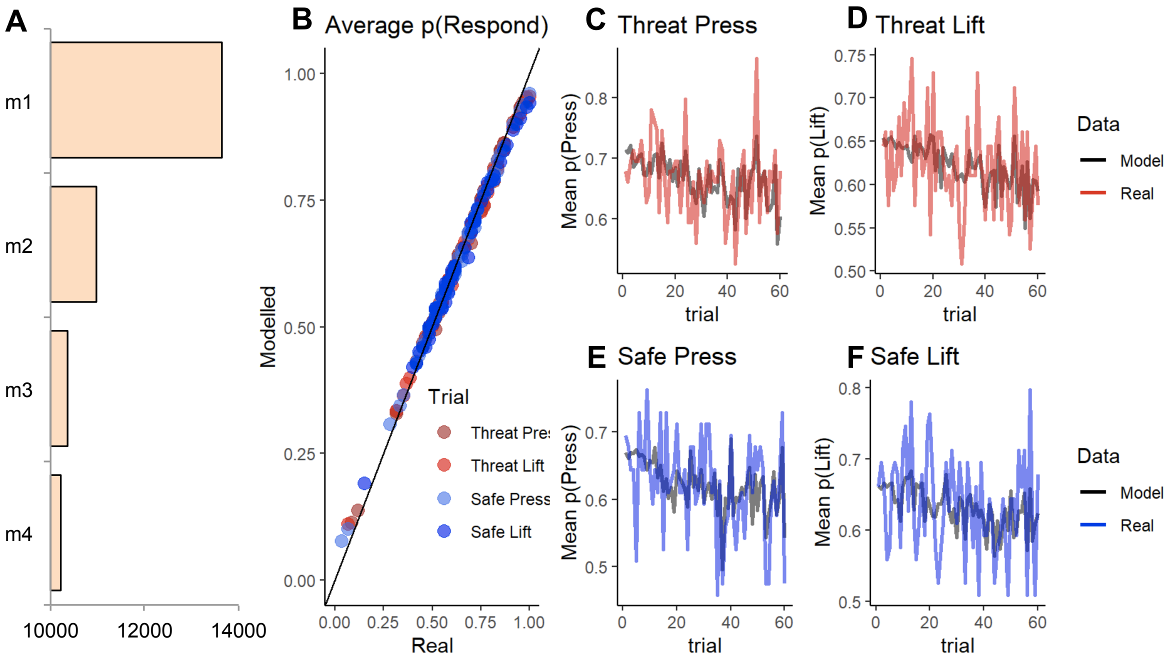

*<u>Figure S2</u> Model comparison revealed that A) m4 (Rescorla Wagner with learning rate (*ep*) + separate reward (*rhoRew*) and punishment (*rhoPun*) sensitivities + noise (*xi*) + action bias (*b*) + pavlovian bias (*pi*)) was the best model. Simulations revealed that this model was able to B) recapitulate the overall average probability of making a response (p(response)) as well as closely match the overall population learning curves for C) Threat Press D) Threat Lift E) Safe Press F) Safe Lift conditions.*

HDI comparisons (Table S1) demonstrated that the only parameter for which there was a credible difference across task conditions (i.e., interaction calculated using difference scores)

was the *xi* (noise) parameter, for which simple effects demonstrated increased behavioural noise in the lift (relative to press) action under the safe condition only (Figure S2).

		xi	***ep***	***b***	***pi***	***rhoRew***	***rhoPun***
Interaction							
**Threat x action**	**LB**	**0.01**	-0.06	-0.13	-0.40	-15.19	-9.88
	**UB**	**0.22**	0.06	1.49	1.03	122.16	9.52
Simple effects							
**Threat -Safe Press**	**LB**	-0.02	-0.04	-0.15	-0.37	-30.05	-8.87
	**UB**	0.06	0.06	1.02	0.43	84.69	4.99
**Threat -Safe Lift**	**LB**	-0.20	-0.02	-0.82	-0.85	-75.84	-8.10
	**UB**	0.00	0.04	0.32	0.30	2.44	5.84
**Press-Lift Threat**	**LB**	-0.04	-0.04	-0.07	-0.40	-3.62	-7.09
	**UB**	0.07	0.06	1.03	0.55	91.09	6.05
**Press-Lift Safe**	**LB**	**-0.19**	-0.02	-0.81	-0.79	-64.72	-7.17
	**UB**	**-0.01**	0.04	0.39	0.27	29.69	7.50

Table S1**:** *Highest Density Interval (HDI) comparisons for all parameters:* xi *(noise),* ep *(learning rate),* b *(action bias),* pi *(Pavlovian bias) and reward (*rhoRew*) and punishment (*rhoPun*) sensitivities. Where the Lower Bound (LB) and Upper Bound (UB) don't overlap zero, there is a credible difference in the given comparison. The only comparison for which this is true (bold) is the threat x action interaction for the xi parameter, which is driven by the press vs. lift comparison under safe conditions.*

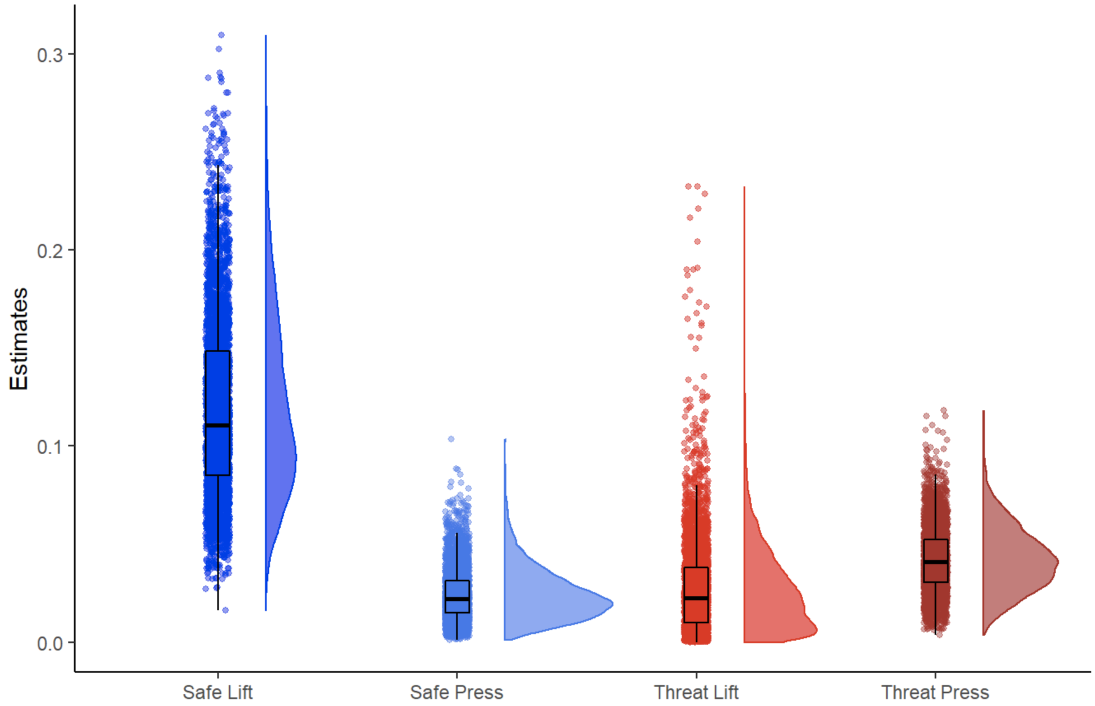

Figure S3: Raincloud plot of the posterior estimates of the xi *(noise) parameter for each of the task conditions reveal that the overall interaction is driven by elevated behavioural noise (i.e., reduced reliance on reinforcement learning as implemented in the model) in the safe lift condition.*
